# Burnout and career satisfaction in neurology

**DOI:** 10.3389/fpubh.2026.1747180

**Published:** 2026-03-12

**Authors:** Kristijonas Puteikis, Rūta Mameniškienė

**Affiliations:** Clinic of Neurology and Neurosurgery, Institute of Clinical Medicine, Faculty of Medicine, Vilnius University, Vilnius, Lithuania

**Keywords:** burnout, dementia, neurologist, occupational health, stroke

## Abstract

**Background:**

Neurology ranks among specialties with the highest burnout rates, yet little is known about neurologists' subjective experiences. We aimed to assess burnout, career satisfaction and associated factors among Lithuanian neurologists, as well as their views on the field's future.

**Methods:**

From June 2024 to March 2025, we conducted a cross-sectional anonymous survey of Lithuanian neurologists. The questionnaire assessed career satisfaction, work–rest balance, and perspectives on neurology. Burnout was measured using the Copenhagen Burnout Inventory. Stepwise linear regression identified associated factors.

**Results:**

Responses were obtained from 111 neurologists (73.0% female; mean age 53.2 ± 12.8 years; mean practice 26.0 ± 14.0 years). Personal, work-related and patient-related symptoms of burnout were present at least sometimes in 44.1%, 30.6%, and 27.0% of respondents, respectively, and commonly linked to insufficient personal time, inadequate leisure, and poor sleep. Regression analyses ($R_{\text{adj}}^{2}$ = 0.11–0.33) identified insufficient personal time and perceived diagnostic limitations as key predictors of burnout. Most participants were satisfied with their role as neurologists (78.4%) and would choose the specialty again (79.3%). While anticipating therapeutic advances, they predicted future declines in healthcare capacity, nursing, and rehabilitation services. Cognitive disorders (74.8%) and sleep–wake disorders (63.1%) were most often cited as emerging areas.

**Conclusion:**

Despite high career satisfaction and optimism regarding therapeutic progress, symptoms of burnout are prevalent among Lithuanian neurologists, driven by suboptimal work–rest balance and systemic limitations. These findings emphasize the need for organizational and policy measures to support physician wellbeing and strengthen healthcare efficiency.

## 1 Introduction

Neurological disorders already represent the third leading cause of disability and premature death in the European Union (EU) and are expected to become increasingly burdensome as the population ages (1). Therefore, a sufficient and empowered neurology workforce has become a public health priority (2, 3). However, neurology may not be especially attractive, as it remains a complex specialty with frequently challenging working conditions. In the United States, it ranked fourth from the bottom for physician satisfaction with personal and family time and third for burnout prevalence (4). A recent systematic review reported that, across studies using the Maslach Burnout Inventory (MBI), around two-thirds (65.9%) of neurologists experience burnout (5). Long working hours, a high patient load, inadequate work-life balance, insufficient support staff, and time-consuming clerical tasks have been cited as major factors contributing to the risk of burnout among neurologists (6, 7). Beyond organizational challenges, compared with many other medical specialties, neurologists face a distinctive combination of prolonged diagnostic uncertainty, limited curative options, and sustained exposure to progressive neurological disability, which amplifies both cognitive and emotional workload. Despite increasing attention toward the strengthening of the neurology workforce among policymakers (2), studies exploring both burnout and other personal aspects of being a neurologist remain relatively scarce, especially in Europe (5). Previous reports often included specific neurologist groups, such as neurointerventionalists (8), cognitive disorder (9), headache (10), or stroke (11, 12) specialists, neurology trainees (13, 14), graduate students (15), or female neurologists (16). Consequently, comprehensive nationwide studies including all practicing neurologists, irrespective of subspecialty, remain limited. The present study addresses these gaps by providing a nationwide assessment of burnout, career satisfaction, and professional perspectives among neurologists across all subspecialties. In addition, it examines how neurologists' experiences within the healthcare system relate to burnout symptoms and explores their views on past achievements and future directions of clinical neurology. A further scoping of the specialists' personal reflections and attitudes in the field of neurology may provide important insights into future improvement in work organization and policies concerning neurological practice.

The objectives of our study were severalfold:

1. To assess the prevalence of burnout and associated factors among Lithuanian neurologists.
2. To determine the level of satisfaction with neurology and associated factors in the same population.
3. To explore the neurologists' perceptions about the current environment and future trends of clinical neurology practice.

## 2 Methods

### 2.1 Study setting

We conducted a cross-sectional survey among Lithuanian neurologists between June 21, 2024 and March 22, 2025. Convenience sampling was used—neurologists attending the 22nd Lithuanian Neurology Summer School (an annual national conference organized by the Lithuanian Association of Neurologists and the Lithuanian Child Neurology Association) were invited to complete anonymous survey forms on paper (later depositing them in a drop box) or using a QR code linking to the respective Google Form online. As students, residents and nurses attended the conference, participants were asked to indicate their professional status in the form. However, only answers by adult and child neurologists were kept for analysis. Additional invitations to complete the same form were sent after the conference using member mailing lists of the Lithuanian Association of Neurologists. The responses were completely untraceable to any particular participant as no personal information was collected. A sample size of 112 was sought to achieve a representation of around 20% of practicing neurologists in Lithuania (the estimate of practicing adult and pediatric neurologists was 560, based on public data provided by the State Accreditation Service for Health Care Activities under the Ministry of Health) (17).

### 2.2 The questionnaire

The translated version of the questionnaire that was used in the study is presented in Supplementary Table 1. The first part of the questionnaire included questions about sociodemographic data and clinical practice.

Participants were then asked to evaluate knowledge, difficulty, confidence, and interest in neurology on a Likert scale from one to five. These items derived from the questionnaire by Schon et al. (18) and were supplemented by two additional questions tapping into the confidence in localizing lesions relying on neurological examination alone and the confidence in treating a patient with a neurological disease.

Subsequently, the questionnaire included *ad hoc* items about the current trends and future prospects of neurology (both from a global and everyday point of view), as well as personal challenges, such as insufficient personal and leisure time or sleep. Respondent satisfaction with their specialty was evaluated based on agreement with the statement “I am happy to be a neurologist” on a five-point Likert scale. Participants were also asked to rate different neurological sub-specialties [categorized in accordance with the suggested domains of the European Training Requirements for Neurology (19)] based on their relevance over the next decade and breakthroughs during the previous decade.

The respondents also completed the Copenhagen Burnout Inventory (CBI), a tool widely used to assess domains of personal, work-related and patient-related burnout (20). Each domain is scored on a scale from 0 to 100 with higher scores representing more frequent symptoms of burnout (8). The questionnaire concluded with the Fatigue Severity Scale (FSS), a seven-item instrument assessing subjective fatigue (the average score across all items was used for analysis) (21).

### 2.3 Statistical analysis

All statistical analyses were done in MS Excel v2507 and IBM SPSS 26. Data normality was assessed using the Kolmogorov-Smirnov test, data distribution histograms and Q-Q plots. The Student's *t* and the Mann-Whitney *U* tests (two independent groups) or the ANOVA and Kruskal-Wallis test (more than two independent groups) were used for comparisons between groups. Pearson's r was used for correlation analysis. Factors associated with different domains of burnout were explored using stepwise multiple linear regression modeling. The threshold for statistical significance was set at *p* < 0.05 and all tests were two-tailed. The target sample size of 112 was deemed to be sufficient to yield a medium effect size of f^2^ = 0.15 with α = 0.05, 1–β = 0.80 in a linear regression model with 8 independent variables (G^*^Power 3.1.9.7).

### 2.4 Ethics

The study was conducted in accordance with the ethical principles of the Declaration of Helsinki and the guidelines of the World Medical Association (WMA). As the study was a fully anonymous survey, it was exempt from approval by the Bioethical Committee. Informed consent was obtained through the survey form.

## 3 Results

### 3.1 General findings

The study sample consisted of 111 neurologists (81, 73.0% female; mean age 53.2 ± 12.8 years), representing around 20% of practicing specialists in Lithuania. The general characteristics of the study sample and their neurology practice are presented in Table 1.

**Table 1:** General characteristics of the study sample.

**Characteristic**	***N*(%) or** **Mean (SD)**
**Sex** ^a^	
Male	29 (26.1)
Female	81 (73.0)
**Age, years**	53.2 (12.8)
**Years of practice**	26.0 (14.0)
**Works in academia (“Yes”)**	25 (22.5)
- If yes, academic experience, years	13.5 (7.9)
**Works in research (“Yes”)**	20 (18.0)
**Specialization**	
Adult neurology	100 (90.1)
Child neurology	11 (9.9)
**Workplace**	
Outpatient clinic	70 (63.1)
Inpatient clinic	60 (54.1)
Emergency department	36 (32.4)
Private practice	31 (27.9)
Nursing and palliative care institution	3 (2.7)
**Place of practice**	
Urban	108 (97.3)
Rural	3 (2.7)
**Expected remaining years of practice** ^a^	
< 5	26 (23.4)
5–10	31 (27.9)
>10	53 (47.7)
**Plans to change the workplace** ^a^	
No	95 (85.6)
Yes, to change the workplace	9 (8.1)
Yes, to work abroad	1 (0.9)
Yes, to change medical speciality	2 (1.8)
Yes, to change profession	3 (2.7)
**Major patient groups consulted**	
Adults	98 (88.3)
Children and adolescents	11 (9.9)
Adults 65 years and older	29 (26.1)
Patients with intellectual disorders	8 (7.2)
Patients with disabilities	16 (14.4)
**Member of the Lithuanian Neurologists'** **Association (“Yes”)**	84 (75.7)
**Member of the European Academy of** **Neurology (“Yes”)**	34 (30.6)
**Member of any specialist neurologist** **society (“Yes”)**	38 (34.2)

^a^ missing *n* = 1, 0.9%.

### 3.2 Burnout

The average personal, work-related and patient-related burnout scores were 43.6 ± 19.1, 39.9 ± 16.1 and 38.9 ± 15.5, respectively, with 49 (44.1%), 34 (30.6%) and 30 (27.0%) considered to have symptoms of burnout at least sometimes in the respective domains (score ≥ 50). There were 56 (50.5%) respondents who had symptoms of burnout at least sometimes in one or more of the domains. The mean FSS score was 3.74 ± 1.22. Personal burnout (*t* = −2.24, *p* = 0.027), work-related burnout (*t* = −2.36, *p* = 0.020), and fatigue (*t* = −3.77, *p* < 0.001) were more expressed in female respondents. Work-related (*r* = −0.20, *p* = 0.038) and patient-related (*r* = −0.22, *p* = 0.022) burnout were inversely associated with age. Self-reported confidence when treating individuals with neurological disorders was inversely associated with personal (*r* = −0.25, *p* = 0.008), work-related (*r* = −0.27, *p* = 0.004), and patient-related (*r* = −0.25, *p* = 0.010) burnout. Personal burnout was higher among individuals working in outpatient clinics (*t* = −2.04, *p* = 0.044) and with pediatric populations (*t* = 3.87, *p* = 0.001). Burnout and fatigue were not associated with work in academia or research, expected years of practice remaining or membership in specialist organizations.

Stepwise linear regression models revealed that burnout was mostly associated with insufficient personal time and perceived inadequate diagnostic capacity (Table 2).

**Table 2:** Stepwise linear regression models with burnout domains as dependent variables.

**Independent variable**	**B and 95% confidence interval**	**Standardized β**	***P* value**
**Personal burnout [*F*_(3, 105)_** **=** **14.66**, ***p*** **<** **0.001**, ${\text{R}}_{\text{adj}}^{2}$ **=** **0.28]**			
“I have enough time for myself” ^a^	−4.59 (−7.87 to −1.32)	−0.28	0.006^*^
“I have enough time for sleep” ^a^	−4.15 (−7.42 to −0.88)	−0.25	0.013^*^
“I have the conditions to effectively diagnose patients' illnesses” ^a^	−3.99 (−7.42 to −0.55)	−0.20	0.023^*^
Constant	44.51 (40.72 to 48.30)	n/a	< 0.001^**^
Excluded variables: age, sex, major patient group—adults, major patient group—children and adolescents, confidence when treating patients with neurological disorders, “I have enough time for my patients” ^a^, “I have enough time for hobbies and leisure” ^a^, “I have the conditions to effectively treat patients' illnesses” ^a^, “I have the conditions to ensure long-term patient care” ^a^			
**Work-related burnout [*F*_(4, 104)_** **=** **14.15**, ***p*** **<** **0.001**, ${\text{R}}_{\text{adj}}^{2}$ **=** **0.33]**			
“I have enough time for hobbies and leisure” ^a^	−3.40 (−6.15 to −0.64)	−0.23	0.016^*^
“I have the conditions to effectively diagnose patients' illnesses” ^a^	−4.39 (−7.17 to −1.60)	−0.25	0.002^*^
“I have enough time for sleep” ^a^	−3.91 (−6.53 to −1.28)	−0.28	0.004^*^
Age	−0.23 (−0.42 to −0.03)	−0.18	0.025^*^
Constant	53.37 (42.33 to 64.41)	n/a	< 0.001^**^
Excluded variables: sex, major patient group—adults, major patient group—children and adolescents, confidence when treating patients with neurological disorders, “I have enough time for my patients” ^a^, “I have enough time for myself” ^a^, “I have the conditions to effectively treat patients' illnesses” ^a^, “I have the conditions to ensure long-term patient care” ^a^			
**Patient-related burnout [*F*_(3, 104)_ = 5.45,** ***p*** **=** **0.002**, ${\text{R}}_{\text{adj}}^{2}$ **=** **0.11]**			
Confidence when treating patients with neurological disorders	−5.37 (−10.17 to −0.58)	−0.21	0.028^*^
“I have enough time for hobbies and leisure” ^a^	−2.71 (−5.28 to −0.14)	−0.19	0.039^*^
Age	−0.22 (−0.44 to −0.003)	−0.18	0.047^*^
Constant	70.41 (49.23 to 91.59)	n/a	< 0.001^**^
Excluded variables: sex, major patient group—adults, major patient group—children and adolescents, “I have enough time for my patients” ^a^, “I have enough time for myself” ^a^, “I have enough time for sleep” ^a^, “I have the conditions to effectively diagnose patients‘ illnesses” ^a^, “I have the conditions to effectively treat patients' illnesses” ^a^, “I have the conditions to ensure long-term patient care” ^a^			

^a^ agreement with the respective statements (five-level Likert scale) is tested as the independent variable. ^*^ *p* < 0.05, ^**^ *p* < 0.001.

### 3.3 Professional satisfaction

Most specialists (85, 76.6%) reported being interested or very interested in neurology. Presented with a hypothetical choice, most neurologists indicated they would still choose neurology at graduation (85, 76.6%) or if they could go back in time (88, 79.3%). The majority (84, 75.7%) would also encourage current medical students to choose neurology.

**Figure 1:**
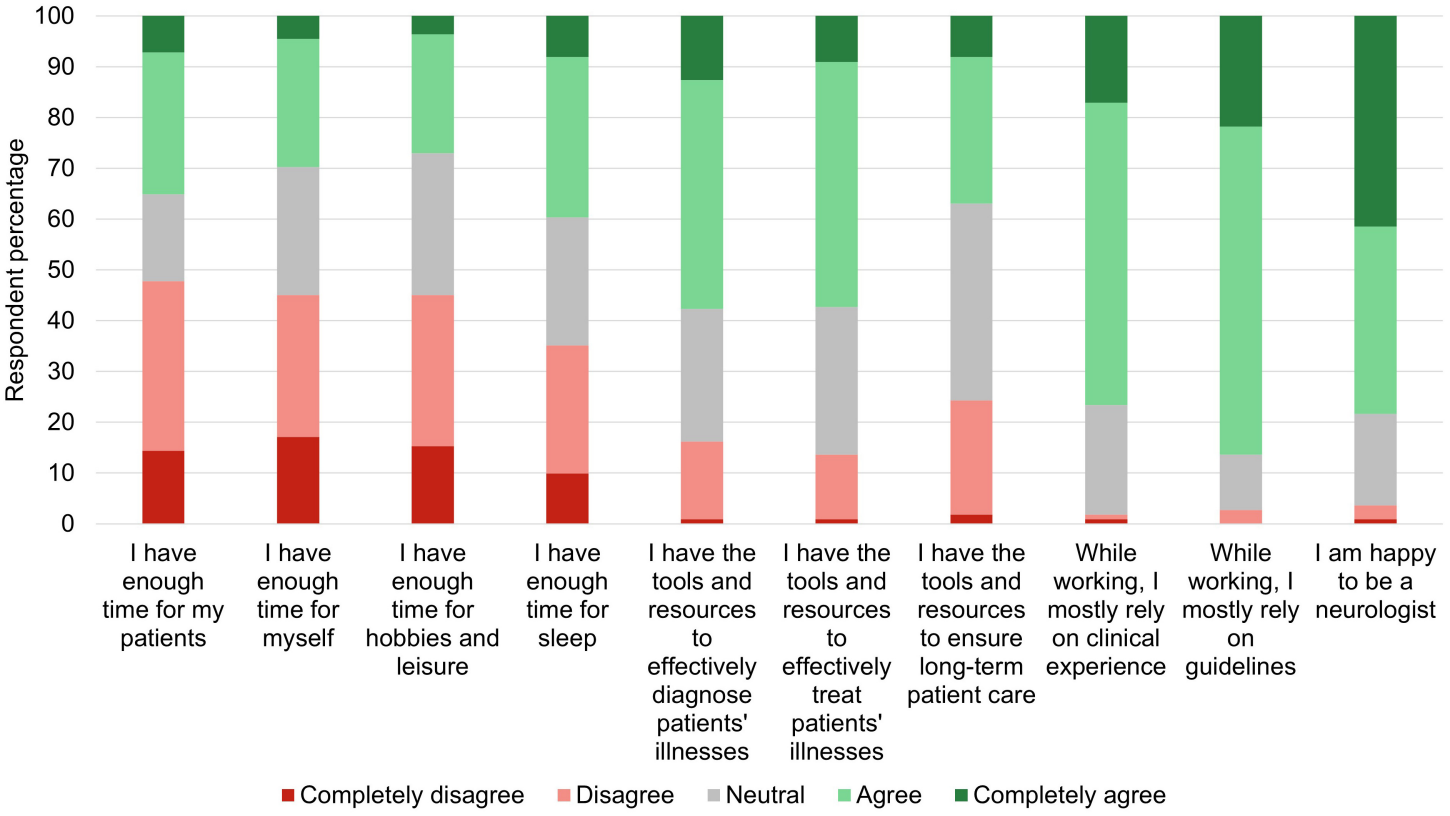
Agreement with statements about personal experiences while working as a neurologist.

While 87 (78.4%) of all participants reported being happy as neurologists, many indicated having insufficient personal and leisure time, as well as insufficient sleep (Figure 1).

The reported satisfaction of being a neurologist was not associated with age, sex, years of clinical experience, specialization (adult/child neurology), or working in academia, but was directly correlated with the perceived capacity to effectively diagnose (*r* = 0.24, *p* = 0.010) and treat (*r* = 0.34, *p* < 0.001) patients, as well as ensure long-term patient care (*r* = 0.25, *p* = 0.008). Neurologists working with inpatients tended to be more satisfied with their specialty (*Z* = 2.08, *p* = 0.037). The satisfaction was inversely related to burnout scores (*r* = −0.22, *p* = 0.021 [personal burnout], *r* = −0.29, *p* = 0.002 [work-related burnout], *r* = −0.31, *p* = 0.001 [patient-related burnout]) but not associated with fatigue or insufficient personal and leisure time (*p* > 0.05).

### 3.4 Perceptions of neurology

**Figure 2:**
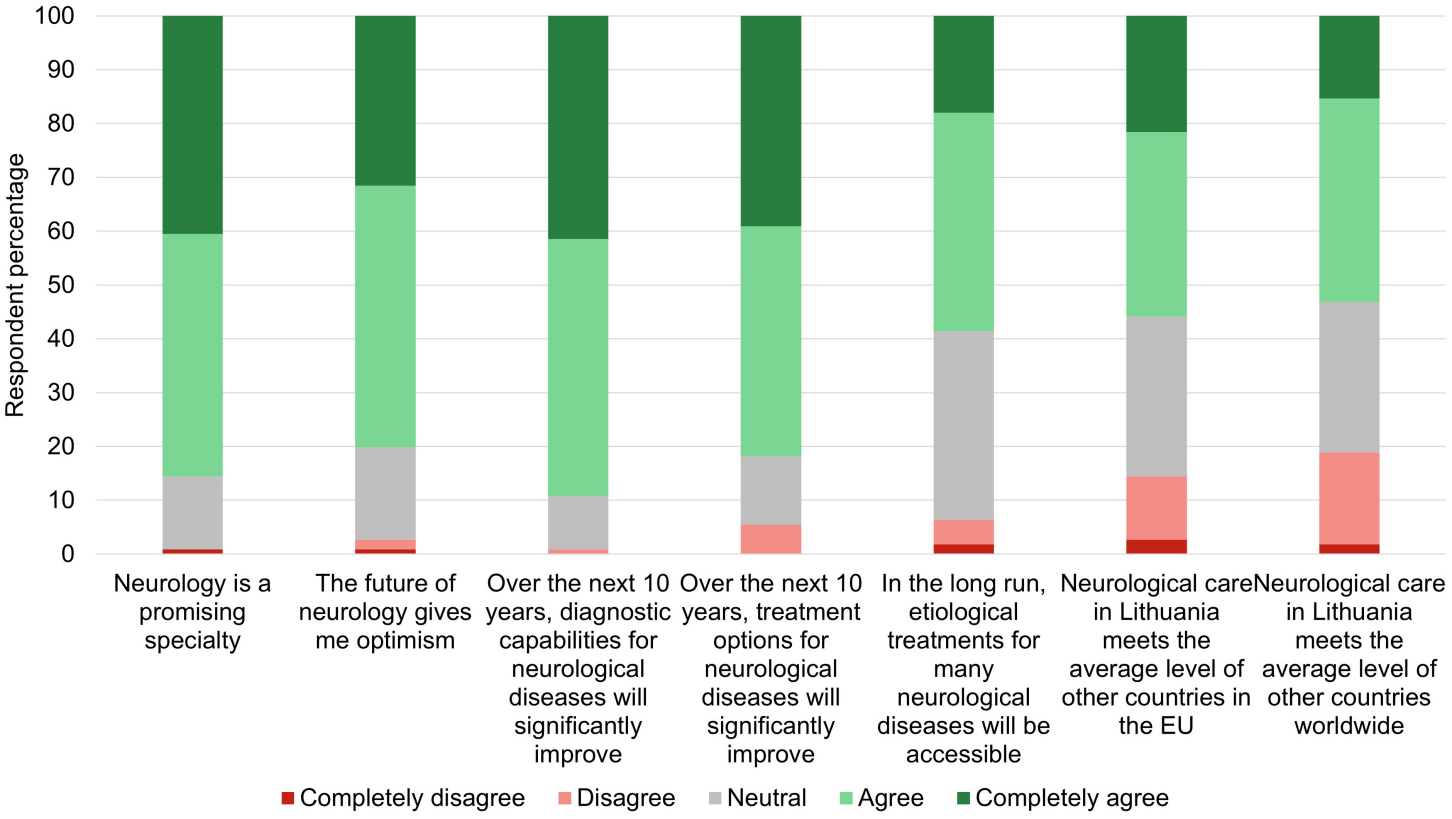
Agreement with statements about the current and future global outlook regarding neurology.

When presented with questions regarding global tendencies of neurology as a medical specialty, respondents tended to have predominantly positive perspectives (Figure 2).

Cognitive disorders and dementias (83, 74.8%) and sleep-wake disorders (70, 63.1%) were most often recognized as neurological disorder groups with emerging relevance over the next decade (Supplementary Figure 1). Most progress in the past 10 years has been acknowledged in the fields of immune-mediated diseases (101, 91.8%) and cerebrovascular disorders (96, 87.3%; Supplementary Figure 2).

**Figure 3:**
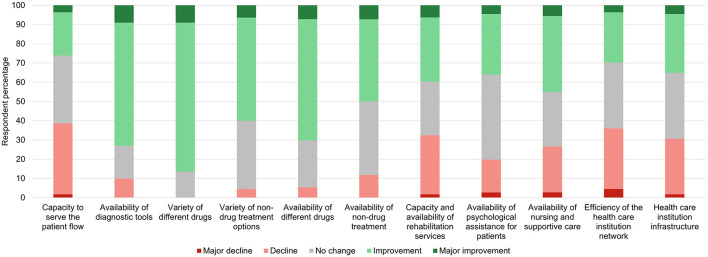
The participants' perspectives regarding expected changes in neurological practice in Lithuania over the next 10 years.

**Figure 4:**
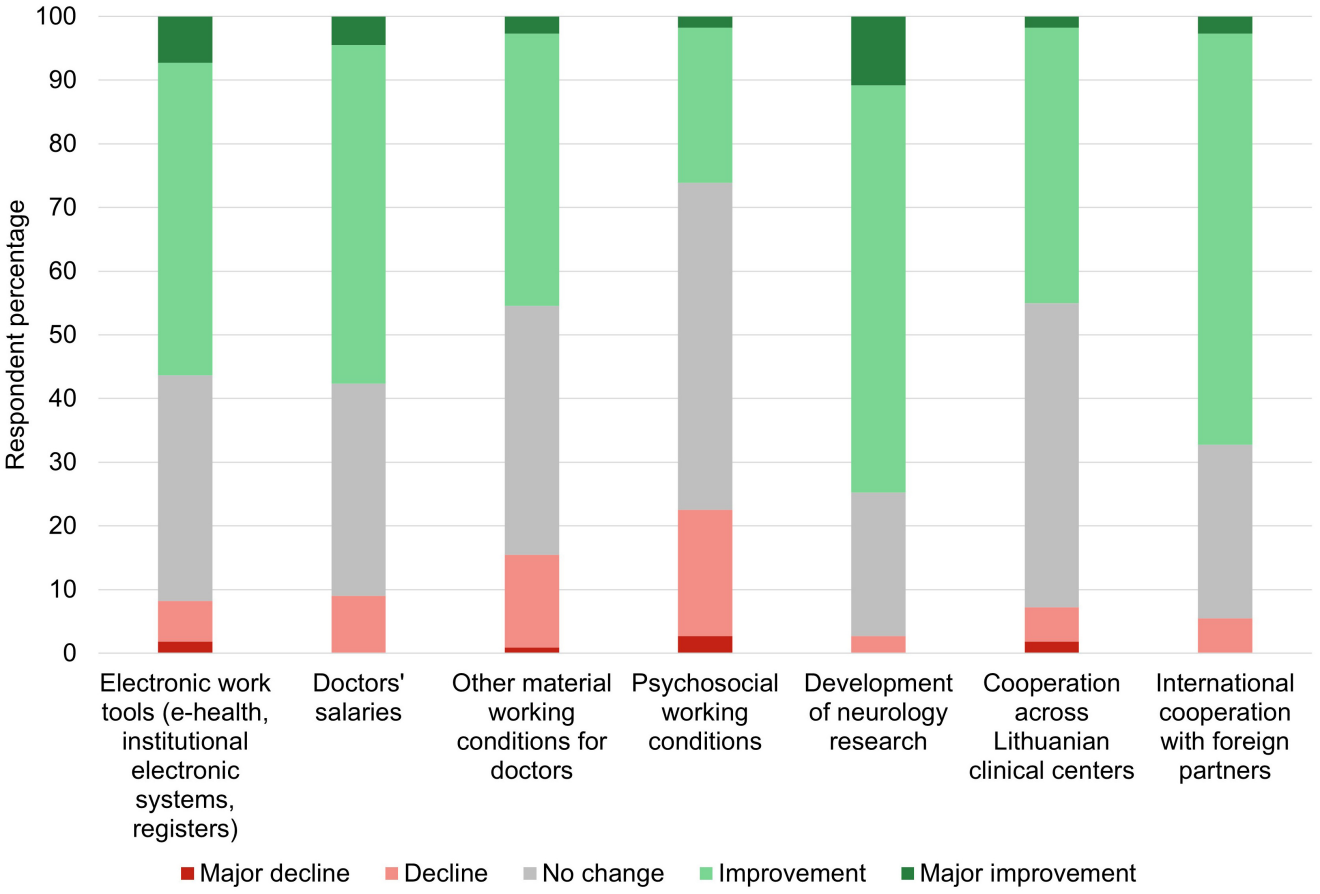
The participants' perspectives regarding expected changes in working conditions in Lithuania over the next 10 years.

Considering future perspectives over the next 10 years in Lithuania, the outlook was mostly positive, with concerns being related to the overall capacity of the healthcare system to serve the patient flow, ensure sufficient nursing and supportive care, and provide rehabilitation services (Figures 3, 4).

## 4 Discussion

We present a nationwide survey exploring burnout, professional satisfaction and general outlook toward the state of their specialty among Lithuanian neurologists.

While the data from our survey revealed that most neurologists are satisfied with their career choice and have positive perceptions concerning the future of neurology, around a third experience symptoms of work-related or patient-related burnout at least sometimes. Moreover, nearly half report experiencing symptoms of personal burnout at least sometimes and having inadequate personal time outside of work. Career choice satisfaction was largely higher than in previous reports (22, 23) and the prevalence of respondents considered to have signs of burnout was lower than recently reported in a meta-analysis summarizing data across China, the U.S., and Brazil (65.9%) (5), but similar to results from a study using the CBI in which burnout among U.S. neurointerventionalists was measured (8). The level of burnout among Lithuanian respondents was associated with inadequate personal, leisure time and insufficient sleep. Such results coincide with previous findings, suggesting that work-life balance is a major factor associated with burnout among neurology specialists (14). It was also more expressed in females and younger specialists. Younger specialists, in need to establish themselves as respected healthcare providers as well as undergo significant changes in their personal lives, such as motherhood or fatherhood, or ensure new financial obligations, face a unique convergence of stressors that can predispose them to burnout. In line with previous findings, symptoms of burnout were more prevalent in female specialists—it has been suggested that gender inequality, such as a lower likelihood of promotion, negative preconceptions on maternal leave and other gender-specific factors may contribute (16). Differential effects of work-related factors (such as the number of hours worked or nights on call) on burnout in female vs. male neurologists have also been noted in a large survey from China (24). Such findings indicate the need for novel institutional (or even nationwide) interventions that would target cultural, administrative, and workload issues in the neurologist community (5). Moreover, the lack of conditions for efficient diagnostics emerged as a distinct factor related to the level of burnout, emphasizing the importance of the availability of diagnostic tools or appropriate referral pathways, as some of technologies (e.g., video electroencephalography, positron emission tomography) may only be available in tertiary centers. Such changes, however, would likely require costly expansion of medical infrastructure, as well as governmental action to optimize the healthcare network.

Among the motivations to conduct the survey, beyond the internationally acknowledged need to better understand and then seek ways to strengthen the neurology workforce, was the recognition of negative attitudes toward neurology as a difficult and complex specialty, a phenomenon widely seen among medical students and junior doctors (25), including those residing in Lithuania (26). This subjective perception makes neurology a less attractive choice for incoming residents and may undermine the more appealing aspects of the specialty. In contrast to undergraduates or physicians from other fields, usually found to have such preconceptions, our survey revealed that most neurologists have positive views of their own specialty: around four in five remain interested in their profession, are happy to be neurologists and would choose it again as a career path. Moreover, the respondents in our study consider neurology to be promising and expect significant improvements in both diagnostics and treatment, including etiological therapies. Such findings suggest that, in contradiction to older notions of neurology being a primarily diagnostic specialty (hence the outdated saying “diagnose and adios”) (18, 27, 28), neurologists envision a future with better treatment options in everyday practice, or even an expanding availability of curative therapies. At least in part, these beliefs may be influenced by rather dramatic changes of the quality of neurological care in Lithuania over the past decade: substantial improvements in stroke outcomes were observed with increased use of reperfusion therapy (29), and many effective new medications became available over the years, including novel immune-based and biological therapies, having a noticeable positive impact on patient populations affected by multiple sclerosis or migraine (30). Respectively, while respondents tend to agree that significant breakthroughs occurred in most areas of neurology, the change in healthcare opportunities for stroke and immune-related neurological disorders is seen as most evident. On the other hand, our findings may also reflect a more general sentiment emerging from the increasingly dynamic global landscape of research and development, as well as favorable regulatory policies, such as approval of humanized monoclonal antibodies targeting β-amyloid by the Food and Drug Administration or rapid advances of gene therapies targeting amyotrophic lateral sclerosis and spinal muscular atrophy (31).

Despite the positive views in terms of future treatment, our study also suggests that Lithuanian neurologists are painfully aware of the dismal population trends (1), and distinguish cognitive disorders and dementia as the predominant challenge of the future. Sleep-wake disorders were recognized as the second most relevant neurological disorder group of the next decade, a notion likely stemming from routine clinical experience that reflects a high (32) and possibly increasing (33, 34) prevalence of this disorder group. Participants in our study also expressed some more pessimistic views when asked to envision the future status of the overall Lithuanian healthcare system, such as its efficiency, infrastructure or its ability to satisfy the increasing demand of neurological services. Given the rapid aging of the Lithuanian population, the relatively low national healthcare expenditure, and the substantial centralization of healthcare services, these concerns may be justified (30, 35, 36). Dedicated communication with policymakers and the emphasis on national commitments to the Intersectoral global action plan on epilepsy and other neurological disorders 2022–2031 (37) may help to recognize neurological care as a top priority.

The current study has several limitations that should be considered. First, the study included about one fifth of practicing specialists in Lithuania, mostly those working in cities. Neurologists less likely to participate in the activities of the Lithuanian Association of Neurologists may hold different views than the ones who completed the survey, introducing the risk of sampling bias (for instance, participants may represent a cohort with higher overall professional motivation and a more positive outlook toward their specialty). The survey was cross-sectional, limiting causal interpretation. Moreover, the study was conducted in a single country, limiting the generalizability of our findings across other healthcare systems. Categorization of burnout levels should be interpreted with caution because of arbitrary cut-off scores that have not been validated in the study population. Finally, several aspects that may be associated with neurologist satisfaction and burnout, such as data on salaries, hours worked, the frequency of night shifts, or subspecialty (11, 14), were not included in the survey. Mental health outcomes, such as symptoms of depression and anxiety, and their relationship with signs of burnout and fatigue should also be explored in future studies (15).

## 5 Conclusions

From a public health perspective, understanding burnout and career satisfaction among neurologists is essential, as workforce wellbeing is likely to influence access to neurological care, continuity of services, and the capacity of healthcare systems to respond to the growing burden of neurological disorders in aging populations. By integrating standardized burnout measures with career satisfaction and professional perspectives in a nationwide neurologist cohort, this study provides a multidimensional view of specialist wellbeing that has rarely been captured in previous research.

Our cross-sectional study shows that neurologists in Lithuania report a lack of personal, leisure, and sleep time, and experience symptoms of burnout from seldom to sometimes. Burnout was more prevalent in female and younger neurologists. Beyond a detrimental work-rest regimen, burnout was associated with the perception of having suboptimal capacity for effective diagnostics of neurological disorders, as well as their treatment and long-term care. On the other hand, our data suggest that most neurologists are satisfied with their career choice and expect positive developments in the field of neurology over the next decade. The satisfaction of being a neurologist was higher among neurologists working at inpatient departments and directly correlated with the perceived capacities to ensure adequate diagnostics, treatment, and long-term care. Future concerns included the expected inability to meet the increasing demand of neurological services and a decline in the efficiency of the healthcare network.

The current study suggests that sufficient tools and resources available for comprehensive patient care, as well as adequate work-life balance are linked to less frequent symptoms of burnout. While neurologists are keen to practice and represent their specialty, according to our data, urgent action is required to (i) improve their everyday working conditions with emphasis on healthy work-rest schedules and (ii) advocate for nationwide optimization of neurological services. Finally, longitudinal studies should explore whether organizational interventions can effectively reduce the symptoms of burnout among neurologists.

## Data Availability

Raw study data is available upon reasonable request from the authors, accompanied by a study plan. Requests to access the datasets should be directed to kristijonas.puteikis@mf.vu.lt.

## Supplementary material

Supplementary Table 1 The questionnaire used in the study (translated from Lithuanian to English).

Supplementary Figure 1 The perceived change in relevance of different neurological subspecialties over the next decade.

Supplementary Figure 2 Respondents' opinions on whether significant breakthroughs in diagnosis and/or treatment were achieved across different neurological subspecialties during the past decade.
