# Supplementary material for: Burnout and career satisfaction in neurology
Source: Front Public Health. 2026 Mar 12;14:1747180. doi: 10.3389/fpubh.2026.1747180 (PMC13018105; doi:10.3389/fpubh.2026.1747180)
Supplement: Supplementary Table 1 — The questionnaire used in the study (translated from Lithuanian to English). [file Supplementary_file_1.docx]

Supplementary Material

**Supplementary Table 1.** The questionnaire used in the study (translated from Lithuanian to English). * – participants that were not adult or pediatric neurologists were excluded from the study.

| **Survey of Lithuanian Neurologists**  **At Vilnius University, we are conducting a study to evaluate the opinions of neurology specialists practicing in Lithuania regarding the neurology specialty, the main achievements in this field in recent years, and the most relevant challenges. We kindly invite you to contribute to our research by completing the questionnaire below.**  All your answers are **anonymous** and **will only be presented in aggregate form** (information that could identify an individual is not collected). We will be very grateful for your sincere answers.  Researchers of the Faculty of Medicine, Vilnius University: Kristijonas Puteikis and Prof. Rūta Mameniškienė.  Contact emails for inquiries: kristijonas.puteikis@mf.vu.lt / [ruta.mameniskiene@santa.lt](mailto:ruta.mameniskiene@santa.lt) | | | | | | | | 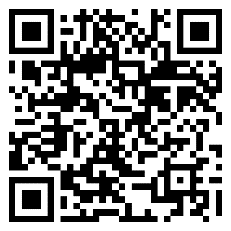**Attention!** For your convenience, the electronic version of the questionnaire is available by scanning the QR code below. | | |  |
| --- | --- | --- | --- | --- | --- | --- | --- | --- | --- | --- | --- |
| **Your sex:** ☐ Male ☐ Female  **Your age:** __________ years  **Your clinical work experience:** _______ years  **Do you work in academia?** ☐ Yes ☐ No  **If yes, academic work experience:** _______ years  **Do you work in research?** ☐ Yes ☐ No | | **Your current position:**  ☐ Neurologist  ☐ Pediatric neurologist  ☐ Neurology resident*  ☐ Pediatric neurology resident*  ☐ Student*  ☐ Nurse*  ☐ Other: _____________* | | | | | | | | |  |
| **Which of the following best describes your workplace (multiple choices possible):**  ☐ Outpatient healthcare facility  ☐ Inpatient healthcare facility  ☐ Emergency department  ☐ Private practice  ☐ Nursing and palliative care institution  ☐ Other (describe): _________________________________ | | **Which of the following best describes your practice location:**  ☐ Urban (City, >3,000 inhabitants)  ☐ Rural (Town, 500–3,000 inhabitants)  ☐ Rural (Village)  **How many more years do you plan to continue your clinical practice?**  ☐ <5 years  ☐ 5–10 years  ☐ >10 years | | | | | | | | |  |
| **Which of the following are the main groups of patients you treat (multiple choices possible):**  ☐ Children and adolescents  ☐ Adults  ☐ People aged 65 and older  ☐ People with intellectual disabilities  ☐ People with disabilities  **Are you a member of the Lithuanian Association of Neurologists?**  ☐ Yes ☐ No  **Are you a member of the European Academy of Neurology?**  ☐ Yes ☐ No | | **Do you plan to change your practice location in the near future?**  ☐ No  ☐ Yes, I plan to work in another institution or care level  ☐ Yes, I plan to work abroad  ☐ Yes, I plan to change medical specialization  ☐ Yes, I plan to change profession  **Are you a member of at least one international specialized neurology association or society?**  ☐ Yes ☐ No | | | | | | | | |  |
| **What is your current level of interest**  **in Neurology?**  □ 1 = Little or no interest  □ 2 = Some interest  □ 3 = Moderate interest  □ 4 = Quite interested  □ 5 = Very interested | | **What is your current level of**  **knowledge of Neurology?**  □ 1 = Little or no knowledge  □ 2 = Some knowledge  □ 3 = Moderate knowledge  □ 4 = Fair knowledge  □ 5 = Great knowledge | | | | | | | | |  |
| **Do you think Neurology is difﬁcult or easy?**  □ 1 = Very difﬁcult  □ 2 = Quite difﬁcult  □ 3 = Moderate  □ 4 = Quite easy  □ 5 = Very easy | | **When you see a patient with neurological complaints in your practice, how do you feel?**  □ 1 = Very uneasy  □ 2 = Uneasy  □ 3 = Averagely competent  □ 4 = Conﬁdent  □ 5 = Very conﬁdent | | | | | | | | |  |
| **How confident do you feel when localizing a neurological lesion based solely on clinical examination results?**  □ 1 = Very uneasy  □ 2 = Uneasy  □ 3 = Averagely competent  □ 4 = Conﬁdent  □ 5 = Very conﬁdent | | **How confident do you feel when treating a patient with a neurological disease?**  □ 1 = Very uneasy  □ 2 = Uneasy  □ 3 = Averagely competent  □ 4 = Conﬁdent  □ 5 = Very conﬁdent | | | | | | | | |  |
| **If you could go back in time and change your choices, would you still choose to become a neurologist?**  ☐ Definitely not ☐ Probably not ☐ I don’t know ☐ Probably yes ☐ Definitely yes | | | | | | | | | | |  |
| **If you were to graduate from medical school this year, would you choose to become a neurologist?**  ☐ Definitely not ☐ Probably not ☐ I don’t know ☐ Probably yes ☐ Definitely yes | | | | | | | | | | |  |
| **Would you encourage medical graduates to become neurologists?**  ☐ Definitely not ☐ Probably not ☐ I don’t know ☐ Probably yes ☐ Definitely yes | | | | | | | | | | |  |
| **Do you agree with the statements below?** | **Strongly disagree** | | **Disagree** | | **Neither agree nor disagree** | | **Agree** | | | **Strongly agree** | |
| Neurology is a promising specialty | ☐ | | ☐ | | ☐ | | ☐ | | | ☐ | |
| The future of neurology gives me optimism | ☐ | | ☐ | | ☐ | | ☐ | | | ☐ | |
| I am happy to be a neurologist | ☐ | | ☐ | | ☐ | | ☐ | | | ☐ | |
| Over the next 10 years, diagnostic capabilities for neurological diseases will significantly improve | ☐ | | ☐ | | ☐ | | ☐ | | | ☐ | |
| Over the next 10 years, treatment options for neurological diseases will significantly improve | ☐ | | ☐ | | ☐ | | ☐ | | | ☐ | |
| In the long run, etiological treatments for many neurological diseases will be accessible | ☐ | | ☐ | | ☐ | | ☐ | | | ☐ | |
| Neurological care in Lithuania meets the average level of other countries in the EU | ☐ | | ☐ | | ☐ | | ☐ | | | ☐ | |
| Neurological care in Lithuania meets the average level of other countries worldwide | ☐ | | ☐ | | ☐ | | ☐ | | | ☐ | |
| I have enough time for my patients | ☐ | | ☐ | | ☐ | | ☐ | | | ☐ | |
| I have enough time for myself | ☐ | | ☐ | | ☐ | | ☐ | | | ☐ | |
| I have enough time for hobbies and leisure | ☐ | | ☐ | | ☐ | | ☐ | | | ☐ | |
| I have enough time for sleep | ☐ | | ☐ | | ☐ | | ☐ | | | ☐ | |
| **While practicing neurology…** |  | |  | |  | |  | | |  | |
| …I have the tools and resources to effectively diagnose patients' illnesses | ☐ | | ☐ | | ☐ | | ☐ | | | ☐ | |
| …I have the tools and resources to effectively treat patients' illnesses | ☐ | | ☐ | | ☐ | | ☐ | | | ☐ | |
| … I have the tools and resources to ensure long-term patient care | ☐ | | ☐ | | ☐ | | ☐ | | | ☐ | |
| ...I mostly rely on clinical experience | ☐ | | ☐ | | ☐ | | ☐ | | | ☐ | |
| ... I mostly rely on Lithuanian or international guidelines | ☐ | | ☐ | | ☐ | | ☐ | | | ☐ | |
| **How do you assess the prospects of neurology in Lithuania over the next 10 years?** | **Major decline** | | | **Decline** | **No change** | **Improvement** | | | **Major improvement** | | |
| Opportunities to serve patient flow | ☐ | | | ☐ | ☐ | ☐ | | | ☐ | | |
| Availability of diagnostic tools | ☐ | | | ☐ | ☐ | ☐ | | | ☐ | | |
| Variety of medication treatment | ☐ | | | ☐ | ☐ | ☐ | | | ☐ | | |
| Variety of non-medication treatment | ☐ | | | ☐ | ☐ | ☐ | | | ☐ | | |
| Availability of medication treatment | ☐ | | | ☐ | ☐ | ☐ | | | ☐ | | |
| Availability of non-medication treatment | ☐ | | | ☐ | ☐ | ☐ | | | ☐ | | |
| Opportunities and availability of rehabilitation services | ☐ | | | ☐ | ☐ | ☐ | | | ☐ | | |
| Availability of psychological assistance for patients | ☐ | | | ☐ | ☐ | ☐ | | | ☐ | | |
| Availability of nursing and supportive care | ☐ | | | ☐ | ☐ | ☐ | | | ☐ | | |
| Efficiency of the health care institution network | ☐ | | | ☐ | ☐ | ☐ | | | ☐ | | |
| Health care institution infrastructure | ☐ | | | ☐ | ☐ | ☐ | | | ☐ | | |
| Electronic work tools (e-health, institutional electronic systems, registers) | ☐ | | | ☐ | ☐ | ☐ | | | ☐ | | |
| Doctors' salaries | ☐ | | | ☐ | ☐ | ☐ | | | ☐ | | |
| Other material working conditions for doctors | ☐ | | | ☐ | ☐ | ☐ | | | ☐ | | |
| Psychosocial working conditions | ☐ | | | ☐ | ☐ | ☐ | | | ☐ | | |
| Development of neurology science | ☐ | | | ☐ | ☐ | ☐ | | | ☐ | | |
| Cooperation between Lithuanian clinical centers | ☐ | | | ☐ | ☐ | ☐ | | | ☐ | | |
| International cooperation with foreign partners | ☐ | | | ☐ | ☐ | ☐ | | | ☐ | | |

**Following items in the questionnaire:**

- Perceived change in relevance of different neurological subspecialties over the next decade.
- Agreement with statements that significant breakthroughs in diagnosis and/or treatment were achieved across different neurological subspecialties during the past decade.
- The Copenhagen Burnout Inventory (CBI) (<https://nfa.dk/vaerktoejer/spoergeskemaer/spoergeskema-til-maaling-af-udbraendthed-cbi/copenhagen-burnout-inventory-cbi/>, Kristensen, T. S., Borritz, M., Villadsen, E., & Christensen, K. B. (2005). The Copenhagen Burnout Inventory: A new tool for the assessment of burnout. Work & Stress, 19(3), 192–207. <https://doi.org/10.1080/02678370500297720>)
- The Fatigue Severity Scale

(Krupp LB, Larocca NG, Muir-Nash J, Steinberg AD. The Fatigue Severity Scale. Application to Patients With Multiple Sclerosis and Systemic Lupus Erythematosus. Arch Neurol 1989;46:1121–3. <https://doi.org/10.1001/archneur.1989.00520460115022>)


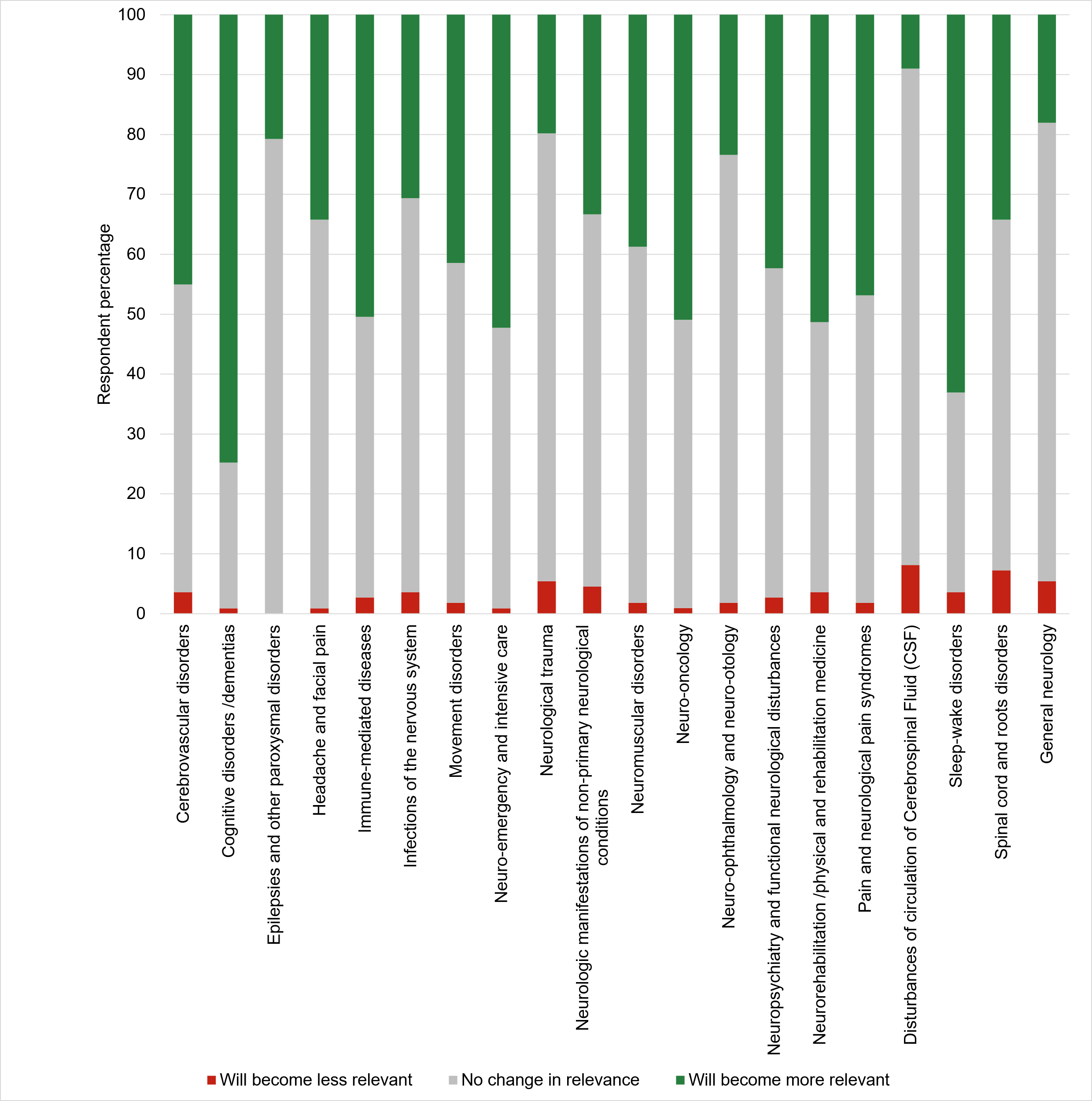


**Supplementary Figure 1.** The perceived change in relevance of different neurological subspecialties over the next decade.

**
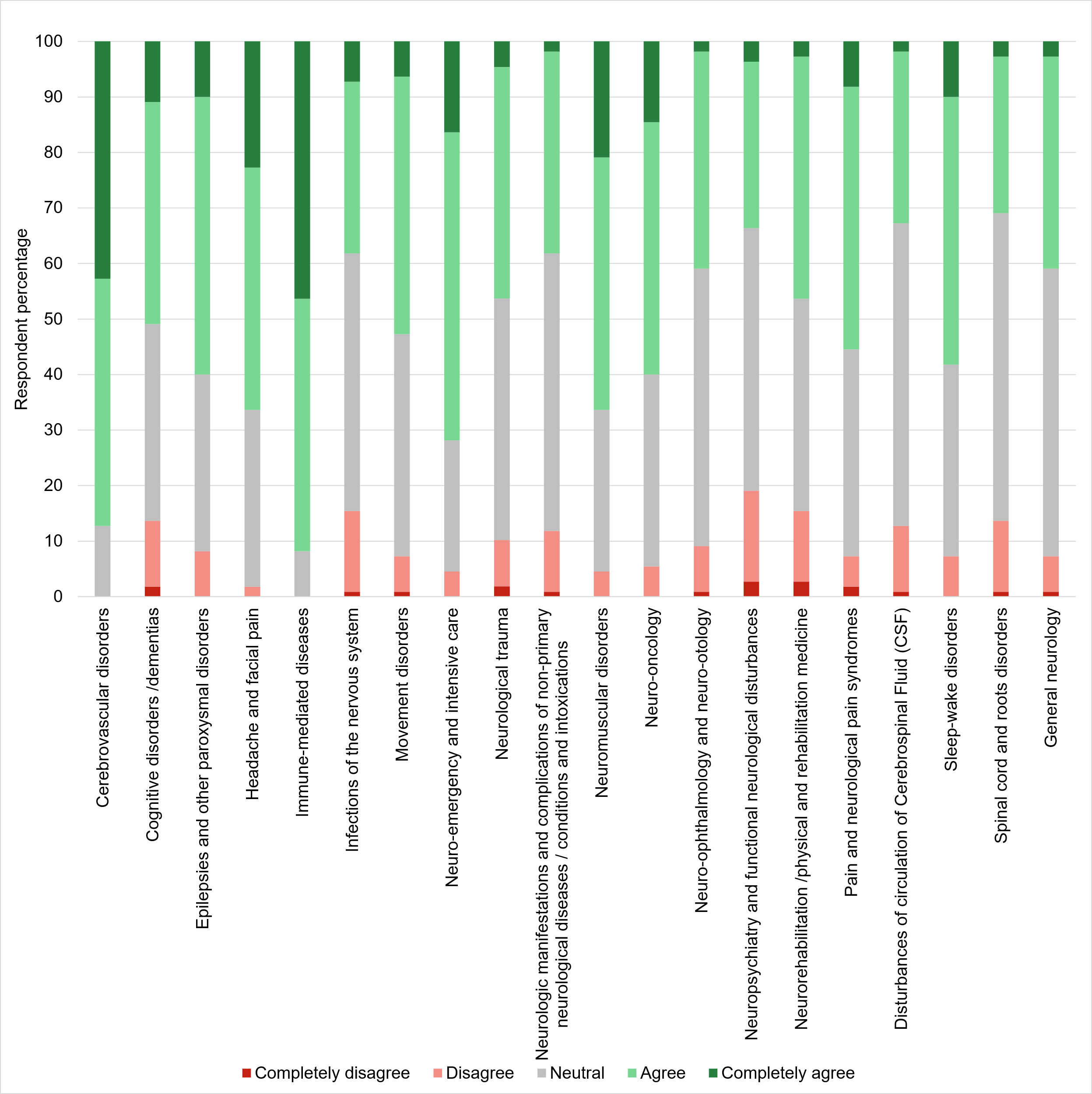
**

**Supplementary Figure 2.** Respondents’ opinions on whether significant breakthroughs in diagnosis and/or treatment were achieved across different neurological subspecialties during the past decade.
